# Immunobiology of primary sclerosing cholangitis

**DOI:** 10.1097/HEP.0000000000001080

**Published:** 2024-09-02

**Authors:** Martin Cornillet, Daniel Geanon, Annika Bergquist, Niklas K. Björkström

**Affiliations:** 1 Department of Medicine Huddinge, Center for Infectious Medicine, Karolinska Institutet, Karolinska University Hospital, Stockholm, Sweden; 2 Unit of Gastroenterology, Department of Medicine Huddinge, Karolinska Institutet, Karolinska University Hospital, Stockholm, Sweden

**Keywords:** cholestatic liver diseases, liver immunology, fibrosis, single-cell methods, autoimmunity

## Abstract

Primary sclerosing cholangitis (PSC) is a chronic inflammatory progressive cholestatic liver disease. Genetic risk factors, the presence of autoantibodies, the strong clinical link with inflammatory bowel disease, and associations with other autoimmune disorders all suggest a pivotal role for the immune system in PSC pathogenesis. In this review, we provide a comprehensive overview of recent immunobiology insights in PSC. A particular emphasis is given to immunological concepts such as tissue residency and knowledge gained from novel technologies, including single-cell RNA sequencing and spatial transcriptomics. This review of the immunobiological landscape of PSC covers major immune cell types known to be enriched in PSC-diseased livers as well as recently described cell types whose biliary localization and contribution to PSC immunopathogenesis remain incompletely described. Finally, we emphasize the importance of time and space in relation to PSC heterogeneity as a key consideration for future studies interrogating the role of the immune system in PSC.

## KEY POINTS

- Using time and space axes, we position the current immune knowledge in PSC and highlight areas to be further explored.
- Most insights originate from the liver of patients after diagnosis and/or fibrotic end-stage disease, where a number of single-cell and spatial immune characterizations have been performed.
- Mononuclear phagocytes, Th17 T cells, and neutrophils close to the bile duct and within the fibrotic areas are some of the most documented cell types where modification of their numbers, location, and function is observed in PSC.
- Studies on intercellular and inter-organ crosstalk, such as the immune-cholangiocyte and gut-liver axes, reveal important propagators of disease activity and warrant further research.

## GENERAL INTRODUCTION

Primary sclerosing cholangitis (PSC) is a chronic inflammatory progressive cholestatic liver disease affecting intrahepatic and/or extrahepatic bile ducts, eventually leading to cirrhosis.^1^ Patients with PSC also stand at high risk of developing biliary tract malignancies.^1^ Many patients with PSC have concomitant inflammatory bowel disease (IBD), and overlap with autoimmune hepatitis occurs.^1,2^ Several genetic risk factors, including a strong human leukocyte antigen (HLA) association, implicate the immune system in PSC.^3^ Beyond this, patients with PSC frequently have autoantibodies, although their role in diagnosis is minor.^4^ All of this together suggests a pivotal role for the immune system, both in the development and progression of PSC. However, it is in this context intriguing that immunomodulatory treatments have limited to no effect on PSC, which contrasts with many other immune-mediated disorders.

Immune cells are abundant in the human liver, which is notably enriched for innate immune cells when compared with peripheral blood.^5,6^ This includes specialized liver macrophages (KCs), natural killer (NK) cells, and unconventional T cells such as CD1d-restricted natural killer T (NKT) cells in mice and mucosal-associated invariant T (MAIT) cells in humans.^5,6^ Beyond this, it is now clear that both fractions of myeloid cells and lymphocytes represent tissue-resident cells adapted to the hepatic microenvironment.^5,6^ Recent technological advancements in single-cell technologies, such as single-cell RNA sequencing (scRNAseq) have improved our understanding of liver immunology by enabling highly granular identification of many discrete subpopulations of immune cells. Indeed, several such single-cell resources now exist for late-stage PSC.^7,8^ Alongside this, the revolution in spatial biology has illustrated that numerous gradients of immune cells exist at the microscopic level in liver lobules^5^ and that the biliary niche represents yet another distinct immune compartment containing specialized myeloid cells and intraepithelial lymphocytes.^9,10^ Also here, spatial resources are now available on end-stage disease PSC livers.^7,11^ These resources and others hold promise for guiding future research regarding the role of the immune system in PSC pathogenesis.

Moving forward, in this review, we will discuss recent immunological insights into PSC pathogenesis, spanning from findings in murine experimental models to translational human studies and implications for immunomodulation in treatment. Recent immunological concepts such as tissue residency and the importance of the spatial microenvironment for cells will be covered in relation to PSC in the upcoming sections that relate to major innate and adaptive immune cell types. As an outlook, unresolved issues will be brought up and open questions for the future will be outlined, with a particular emphasis on time and space in contextualizing PSC disease heterogeneity.

## IMMUNE CELLS IN PSC

**FIGURE 1:**
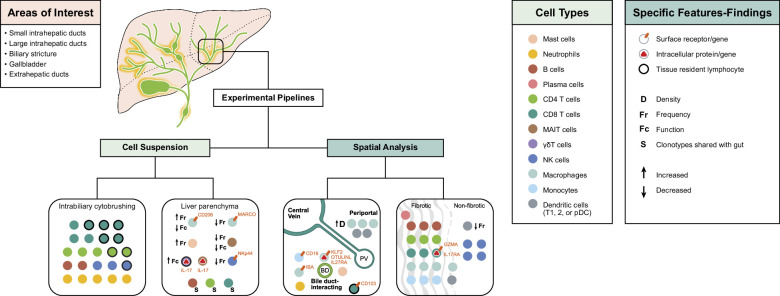
Overview of PSC immunobiology. Summary of the immune features from PSC livers is presented in extended circles depending on the techniques used. These mainly arise from studies on parenchyma of advanced PSC livers, whereas less is known regarding extrahepatic bile duct, intrahepatic and extrahepatic strictures, gallbladder, or intrahepatic small duct areas as indicated by the areas of interest box. Immune cells and specific features/findings are depicted by colored circles, symbols, and letters. Analysis of cell suspensions shows the immune composition of the biliary tree (cytobrushing) and the global modifications of the immune system in the liver parenchyma. Spatial analysis highlights differences within the liver based on a central to portal (PV) axis, bile-duct interacting (BD) cells, as well as in fibrotic versus nonfibrotic areas. Abbreviations: BD, bile duct; PSC, primary sclerosing cholangitis; PV, portal vein.

In the following sections, current knowledge and possible roles for different innate and adaptive immune cells in PSC pathogenesis will be discussed, incorporating findings from both murine experimental systems and human translational studies (Figure 1).

### Neutrophils

Neutrophils, the most abundant immune cell type in the human blood, are essential mediators of inflammatory innate immune responses. Most notably known for their role in resolving acute infection through phagocytosis and degranulation, neutrophils also shape the adaptive immune responses through chemokine and inflammatory cytokine production.^12^ While neutrophil accumulation in tissues is essential for combating bacterial and viral infections, neutrophils have been shown to contribute to the immunopathology of many chronic inflammatory conditions.^13,14^ Indeed, elevated levels of neutrophils in the bile ducts of patients with PSC, both at earlier and later disease stages, represent an important immunological hallmark of disease pathology.^9^ In the Mdr2^−/−^ mouse model, a commonly used mouse model for PSC pathogenesis studies where bile secretion is affected, neutrophils have been shown to be important propagators of biliary inflammation.^15^ Immunofluorescence staining of liver biopsies from Mdr2^−/−^ mice revealed increased presence of activated neutrophils and formation of neutrophil extracellular traps (NETosis) compared with wild-type mice.^15^ In this model, chemical inhibition of neutrophil elastase proved hepatoprotective, as indicated by decreased serum levels of ALP and ALT.^15^ Regarding neutrophil trafficking, elevated C-C motif chemokine ligand (CCL)24 expression from cholangiocytes and liver macrophages in Mdr2^−/−^ mice has been shown to promote neutrophil recruitment to the inflamed bile ducts.^16^ Subsequently, the use of a CCL24-neutralizing antibody abrogated neutrophil recruitment to the bile ducts and mitigated liver fibrosis.^16^ In the human setting, CCL24 was found to be enriched in liver biopsies of patients with PSC.^16^ In addition, CCL24 serum levels correlated with Enhanced Liver Fibrosis score.^16,17^ IL-8, a chemokine that attracts neutrophils through CXCR1/2, was also found to be elevated in the bile of patients with PSC and to correlate with the number of neutrophils in bile ducts of patients with PSC.^9,18^ Together, these cytokines and chemokines represent important modulators of neutrophilic infiltration in PSC, and blocking the accumulation of neutrophils in the bile ducts of patients with PSC represents a promising therapeutic avenue for mitigating biliary inflammation.^19^

Pathogenic IL-17 signaling has been implicated as a propagator of biliary inflammation in PSC (Th17 responses are discussed more in subsequent sections covering T cells),^8,20,21^ yet the specific role of neutrophils in driving IL-17 production in PSC remains to be elucidated. Neutrophils are capable of IL-17 production and have been shown to produce IL-17 in different contexts.^22,23^ More importantly, neutrophils have been shown to skew T-cell differentiation toward a Th17 phenotype through alarmins and histones contained within NETs,^12^ as well as through IL-23 production.^24^ Alongside this, studies in mice leveraging intravital microscopy have demonstrated that IL-17 (synergistically with TNF) mediates neutrophil rolling and endothelial transmigration through selective upregulation of E-selectin and expression of neutrophilic chemokines CXCL1, CXCL2, and CXCL5 by endothelial cells.^25^ Taken together, these findings suggest a positive feedback loop in which neutrophils are recruited to inflamed bile ducts, thereby skewing resident T cells toward a Th17 phenotype, which further propagates neutrophil recruitment to the inflamed bile duct. Therefore, the extent to which this occurs in PSC patients represents an important direction for research regarding the immunopathogenesis of PSC.

Although neutrophils are short-lived, their heterogeneity has recently been explored in the context of infection and cancer. Indeed, these studies have uncovered vast cellular heterogeneity upon emergency granulopoiesis or when neutrophils enter tissues.^26–28^ Immature neutrophils can display immunosuppressive effector functions relative to their mature counterparts, which can display a more proinflammatory phenotype.^29,30^ While Mdr2^−/−^ mice primarily contained mature and activated CXCR2^high^CXCR4^low^CD62L^low^ neutrophils,^15^ limited studies have been conducted to assess biliary neutrophil heterogeneity in humans. One recent study investigating neutrophil heterogeneity in chronic liver diseases uncovered a subset of CXCR4^hi^ CD62L^low^ aged neutrophils termed ductular reaction–associated neutrophils or “DRANs.”^31^ These cells accumulate at the site of ductular reaction in patients and display a proinflammatory transcriptomic profile in 3,5-diethoxycarbonyl-1,4-dihydrocollidine (DDC)-fed mice.^31^ Lastly, depletion of these neutrophils (with either an anti-Ly6G antibody or a CXCR1/2 antagonist) in the DDC-fed mouse liver mitigated ductular reaction, fibrosis, and angiogenesis, demonstrating that these neutrophils are important propagators of ductular reaction in chronic liver diseases.^31^ Thus, further research into the phenotypic and functional heterogeneity of biliary neutrophils in patients with PSC is warranted. Beyond directly studying neutrophils in relation to PSC, it will also be important to determine the impact bacterial cholangitis, a feature of patients with PSC, has as a secondary late phenomenon on neutrophil infiltration and function.

### Mononuclear phagocytes

The mononuclear phagocyte system mainly comprises monocytes (blood circulating), macrophages/KCs (tissue-resident), and dendritic cells (DCs), all of which display phenotypic and functional heterogeneity.^32^ Collectively, these cells contribute to tissue repair and resolution of inflammation, especially through the clearance of pathogens and cell debris involving phagocytosis, but can also cause inflammation.^32^ They further play a pivotal role due to their specific secretome, interactome, and capacity to perform antigen presentation that can modulate both the innate and adaptive compartments of the broader immune system.^32^ Monocytes from the blood of patients with PSC have been reported to display an increased capacity to secrete IL-1β and IL-6 upon microbial stimulation as compared with healthy individuals with consequences for T-cell responses (discussed below).^33^ Circulating monocytes patrol and infiltrate the inflamed liver and have the capacity to differentiate and become tissue macrophages/KCs.^34^ In this context, CD16+ monocytes (receptor binding to the Fc portion of IgG antibodies) were seen preferentially accumulated in the liver of patients with PSC^35^ and located around bile ducts.^33^ Spatial transcriptomics (ST) and scRNAseq studies have recently provided further details on the transcriptional profiles and locations of monocyte and macrophage subsets in PSC. A first ST study reported an increased number of monocytes in fibrotic regions associated with a modification of the tissue monocyte subsets, suggesting a preferential infiltration of fibrotic areas with higher frequencies of tissue monocytes (*IL17RA+S100A8+FCER1G+*) and KCs (*VCAM1+SDC3+*).^11^ A second ST study further described the infiltrate around the biliary tract, showing an increased density of inflammatory macrophages (expressing *CD209*, *CCL4*, *IL1B*, and *FCGR3A*).^36^ A third study combining ST and scRNAseq reported a general increase in macrophage diversity as well as several PSC-specific clusters predominantly of monocyte-like macrophages. Upregulation of *INFGR2*, *CXCL16*, and *CCR1* was noticed in PSC. More specifically, macrophages had increased expression of inhibitory molecules *KLF2, OTULINL*, and *IL27RA*, whereas monocyte-like macrophages were described to display a “fibrosis-associated phenotype” with the upregulation of *LGALS3*, *SPP13*, and *ADA2*. ST indicated an enrichment of these cells around cholangiocytes in fibrotic regions, whereas KCs were localized outside further completing the picture of cell subsets associated and possibly contributing to the fibrotic processes. Beyond phenotyping, macrophages were further described in the same study as hypofunctional with a decreased response to lipopolysaccharide (assessed by CD45+CD68+CD206+TNF+ cells using flow cytometry).^7^ Besides ST and scRNAseq studies, immunohistochemistry using the pan-macrophage marker CD68 showed an accumulation of such cells in both parenchymal and peribiliary areas of PSC livers as compared with control livers.^37^ Finally, an increased percentage of CD206+ macrophages was found in PSC livers in comparison with controls^7,35^ fewer MARCO+ macrophages (macrophage receptor with collagenous structure, a scavenger receptor), although the total number of macrophages was comparable.^38,39^ Altogether, these data suggest that spatial diversity of mononuclear phagocytes within the PSC livers might be globally affected rather than particular types of cells being excluded from specific areas. Along those lines, an increased macrophage diversity was reported in PSC together with the identification of several PSC-specific clusters of monocyte-like cells.^7^ These studies suggest that monocytes and macrophages from fibrotic areas have the potential to both secrete and respond differently to the microenvironment and that this might contribute to the propagation of the immunofibrogenic process.

Several animal models have described the importance of macrophages in shaping cholestatic and fibrotic processes. Of note, the importance of IBA+ macrophages seen in humans was also confirmed in the Mdr2^−/−^ model as they were described to originate from infiltrating monocytes, increased in number as compared with wild type, and located close to the bile duct.^40^ Furthermore, inhibition of the C-C motif chemokine receptor (CCR)2/CCL2 axis (using CCR2 agonist and Ccr2^−/−^ mice), a major monocytic recruitment pathway, attenuated biliary injury in the BV6 (acute sclerosing cholangitis following intrabiliary instillation of BV6 which sensitizes for apoptosis) and Mdr2^−/−^ models^37^ and deletion of farnesoid X receptor in myeloid cells (Mrd2^−/−^ mice) led to the identification of a macrophage-Th1/17 axis associated with a sclerosing cholangitis phenotype. Finally, the Trem2-expressing monocytes/macrophages subset,^41,42^ as well as the expression of Arid3^43^ and CCL24^16^ in macrophages, were identified as important in driving the severity of cholestatic models. However, conditional depletion of KCs (using Clec4f^DTR^ transgenic mice) before cholestasis induction by DDC diet and common bile duct ligation had no effect on disease outcome, thus contesting these cells as major drivers of early pathogenesis.^44^ Overall, data from mouse models mostly suggest a causative role of these cells in driving fibrosis development in the context of cholestasis, although considerable differences exist in the composition of the mononuclear phagocyte system across species.

Beyond monocytes and macrophages, DCs have been studied to some extent in PSC. They comprised several subsets, such as plasmacytoid DC, and 3 main conventional DC subsets with distinct phenotypes and functions.^45,46^ DC density has been reported as increased in the portal area of patients with PSC as compared with liver disease controls, and mouse models of cholangitis reported an increase of intrahepatic conventional DC2 (cDC2) but not cDC1 or plasmacytoid DC.^47^ In humans, deconvolution of ST data from livers of patients with PSC indicated a decreased proportion of cDC1 (not cDC2) in fibrotic regions as opposed to the parenchyma.^11^ In contrast, the deconvolution of DCs from RNAseq of PSC livers suggested an increase in conventional DCs with PSC disease stages.^40^ Finally, the assessment of DCs in PSC livers using scRNAseq identified these cells as one of the disease-associated cell types (cell subset frequency increased in PSC, both plasmacytoid and conventional) as compared with normal livers.^7^ However, compared with our knowledge of monocytes and macrophages, much less is known about DCs in PSC. If and how they crosstalk with conventional and unconventional T cells or B cells warrants further studies. Similarly, priming events taking place either in the gut or in liver/bile duct-draining lymph nodes remain relevant to assess.

In summary, the last decade has provided insights into the immune infiltrate of fibrotic PSC livers, and mononuclear phagocytes have repeatedly been identified as key cell types in shaping liver fibrosis. The most recent studies have further revealed mononuclear phagocyte transcriptional profiles possibly imperative to inflammation and fibrosis observed in PSC. As these detailed phenotypes should be further confirmed and their functional implication better explored, whether these are a consequence of the liver damage or driver of the disease is currently unknown. Comparisons of PSC myeloid cell transcriptomic signatures to those of fibrotic controls from other liver diseases, alongside mechanistic studies exploring the role of such signatures will help us better understand the involvement of mononuclear phagocytes in PSC pathogenesis.

### Mast cells

The role of mast cells in the progression of liver diseases, including cholestatic diseases, has recently been reviewed and involves these cells possessing the capacity to release a broad range of molecules acting on immune and parenchymal cells such as cytokines (TNF, IL-1β, IL-6, IL-8, and TGF-β), peptidases, and other mediators such as histamine, leukotriene, and prostaglandins.^48,49^ Mast cell infiltration has been reported in the livers of patients with PSC by immunochemistry near bile ducts^50^ and portal tracts.^51^ Mast cells have further been identified as one of the disease-associated cell types using scRNAseq of cirrhotic PSC livers compared with normal livers.^7^ However, most of the current knowledge on these cells comes from animal models where their role in cholangitis and fibrosis has been described. Using the Mdr2^−/−^ model, inhibition of mast cell–derived histamine,^52^ antagonism of histamine receptors,^53^ inhibition of the apical sodium BA transporter (expressed on mast cells),^54^ treatment with ursodeoxycholic acid (UDCA),^55^ or cromolyn sodium^50^ all support a role for mast cells in cholangitis and fibrosis progression.

### Innate lymphoid cells

Innate lymphoid cells (ILCs) are immune cells enriched in mucosal barriers defined by their rapid production of effector cytokines in response to epithelial stress signals and other myeloid-derived cytokines.^56–59^ From an effector standpoint, ILCs mirror conventional T helper cell subsets in their cytokine production yet notably lack T-cell receptors and, therefore, antigen specificity.^56–59^ Canonical ILC subsets include NK cells, ILC1s, intraepithelial ILC1s, ILC2s, natural cytotoxicity receptor–negative ILC3s, and natural cytotoxicity receptor–positive ILC3s.^56–59^ In contrast to peripheral blood, where terminally differentiated CD56^dim^ NK cells are abundant, CD56^bright^ NK cells and other ILC subsets predominate in tissues.^6,60^ In humans, CD56^bright^ NK cells expressing CD69, CXCR6, and the transcription factor Eomes, termed liver resident NK cells, represent the most abundant liver ILC subset, constituting up to 35% of all lymphocytes in the healthy human liver.^61–63^ Work investigating peripheral blood NK cells in patients with PSC found that terminally differentiated CD57+ CD56^dim^ NK cells were decreased relative to healthy controls.^64^ In addition, the reduction of CD57 on peripheral CD56^dim^ NK cells correlated with serum bilirubin and ALP levels.^64^ However, whether these cells traffic to bile ducts remains to be determined. Recently, an HLA-DP risk haplotype has been described in PSC directly related to NK-cell functionality.^65^ After confirming HLA-DPA1*02:01-DPB1*01:01 binding to NKp44 (an activating NK-cell receptor) with a bead-based assay, it was shown that plate-bound HLA-DPA1*02:01-DPB1*01:01 induced significantly higher degranulation of primary NKp44+ NK cells in vitro.^65^ This activation was specifically inhibited through an anti-NKp44 blocking antibody, uncovering a possible novel inflammatory axis in PSC.^65^ Of note, while HLA-DP expression was increased in PSC liver explants, the frequency of NKp44+ NK cells was decreased in these livers relative to nonautoimmune liver disease controls.^65^ Therefore, the extent to which NK cells localize to inflamed bile ducts in PSC, and whether they contribute to immunopathogenesis, remains to be elucidated. Along similar lines, little is known about non-NK ILCs in PSC, both in peripheral blood and in the inflamed liver and biliary tract system.

### Unconventional T cells

While granulocytes likely represent transiently infiltrating effector cells responding to inflammation, human bile ducts are populated by tissue-resident lymphocytes that likely reside in situ for many years at a time, similar to other organ systems.^5,63^ Conventional tissue-resident memory T cells (TRMs) (discussed more in detail in the upcoming section) have been shown to be the most abundant lymphocyte population in human bile ducts.^9^ However, a plethora of other innate lymphocytes and unconventional T cells have been described in human bile ducts, with their abundance and phenotype altered in the context of PSC.

MAIT cells, NKT cells, and gamma delta T (γδT) cells represent 3 classes of unconventional T cells identified in the human bile ducts, with each of these subsets responding to different classes of antigens. MAIT cells, prevalent in the human liver, recognize microbially derived vitamin metabolites presented by MR1, an MHC class I-like molecule, and exhibit potent Th1 and Th17 responses.^66^ NKT cells, prevalent in the murine liver, are restricted by lipid antigens presented by CD1d and are also capable of Th1 and Th17 responses.^67^ Finally, γδT cells can recognize a host of different antigens, including phosphoantigens and stress-induced ligands such as MICA/MICB.^68,69^ In the context of PSC, circulating MAIT cells were found to be substantially reduced in abundance relative to control patients.^70^ While they displayed a highly activated phenotype ex vivo, in vitro analysis revealed diminished interferon (IFN)-γ and TNF responses compared with MAIT cells from healthy controls.^70^ Indeed, subsequent in vitro experiments coculturing bile from patients with PSC with peripheral blood mononuclear cells, with and without MR1-blockade, demonstrated that MAIT cell activating antigens are present in the bile of patients with PSC.^71^ In this context, recent work has suggested that sulfated bile acids represent a host-ligand for MAIT cells.^72^ However, it remains to be determined if those bile acids are dysregulated in PSC. Furthermore, recent work outside of the liver and PSC suggests that MAIT cells not only contribute to proinflammatory processes but can also have an active role in wound healing.^73^ Future studies on such reparative (or fibrosis-causing) processes are warranted in PSC.

Similar to that of MAIT cells, bile from patients with PSC has also been shown to contain lipid antigens activating NKT cells.^74^ Diluted bile samples from patients with PSC added to murine NKT cells induced IL-2 production, and this activation was inhibited by blocking CD1d.^74^ To further investigate the role of NKT cells in PSC immunopathology, researchers used the NKT cell activating agent oxazolone in a murine model.^75^ Oxazalone injection into the bile duct increased liver fibrosis in a CD1d-dependent manner since CD1d^−/−^ mice were protected against weight loss, increased ALT, and inflammation.^75^ While the described studies concerning NKT cells have implicated their importance in PSC immunopathology, their abundance and possible deregulation in the human liver remain to be detailed. Regarding γδT cells, Mdr2^−/−^ mice have been shown to have increased levels of IL-17+ γδT cells in the liver compared with controls.^76^ Alongside this, i.v. injection with an antibody directed against the γδT-cell receptor reduced fibrosis in Mdr2^−/−^ mice with a corresponding decrease of IL-17 detected in the serum.^76^ In humans, intrahepatic γδT cells from patients with PSC could produce IL-17 upon stimulation, in contrast to γδT cells from patients with other liver diseases.^76^ This suggests that γδT cells might be part of the dysregulated Th17 axis in PSC.

In summary, the liver is enriched in unconventional T cells, and they could represent important contributors to inflammation and fibrosis progression. Species differences exist between mice and humans (high numbers of NKT in mice but low MAIT and vice versa in humans and more potent Th17 responses in mice), and consolidation of findings from murine models in translational settings is therefore of importance.

### Conventional T cells

Many of the identified PSC susceptibility genes can be related to the function of adaptive lymphocytes and T cells in particular (eg, the strong HLA association as well as *CTLA4/CD28*, *IL2/IL21*, *IL2RA*, *SIK2*, *PTPN2*, *BACH2/MIR4464*, *HDAC7*, *SH2B3*, *MMEL1/TNFRSF14*, *CCL20*, *FOXP1*, *CD226*, *PRKD2*, and *CCDC88B*).^1,77,78^ Yet many of the PSC risk-variants reside in noncoding regions of the genome, making it harder to link them directly to pathogenesis. To address this, a recent study made use of cell-type–specific epigenomic data and assessed if the identified SNPs could contribute to epigenetic dysregulation and found an enrichment of risk-variants within immune-cell–specific enhancers involved in T-cell responses.^79^ An additional link between genetics, T cells, and PSC came from the identification of a family with autosomal dominant PSC having a missense-mutation in *SEMA4D* (semaphorin 4D) encoding CD100.^80^ Biliary-infiltrating T cells were further shown to be the main CD100-expressing cells, and the mutation boosted Th17 differentiation.^20^ More support for a dysregulated Th17-axis in PSC comes from both human translational studies and murine models. A large study relying on brush samples from endoscopic retrograde cholangiopancreatography revealed an enrichment of TRMs with the capacity to produce IL-17 in large bile ducts of both patients with early and end-stage PSC.^9^ The increase of biliary TRMs in PSC has been confirmed by subsequent work as well as positively associated with disease severity.^81^ A role for the transcription factor Runx3 in driving this phenotype has also been suggested.^81^ Furthermore, scRNAseq of cirrhotic PSC compared with control livers have identified an enrichment for naïve-like CD4 T cells epigenetically primed for Th17 differentiation in advanced PSC.^8^ The PSC risk variant associated with the *BACH2/MIR4464* loci was also recently shown to increase the likelihood of naïve T cells polarizing toward a proinflammatory phenotype.^82^ Spatial assessment of cirrhotic PSC livers revealed enrichment of both Th17 cells and cytotoxic T cells in fibrotic compared with parenchymal areas.^11^ Beyond Th17, studies of Mdr2^−/−^ mice have also suggested a role for Th1-responses with enrichment of intrahepatic cytotoxic T cells and NK cells as well as exaggerated IFN-γ production and amelioration of disease upon depletion of T cells or cytotoxic molecules.^83–85^

The dysregulated Th17-axis has been linked to proinflammatory cytokines and diminished T regulatory cell (Treg) function. As discussed above, monocytes respond to microbial dysbiosis in PSC with enhanced production of IL-1β and IL-6 that subsequently promote Th17 cells and IFN-γ responses from T cells.^33,86^ IL-6 signaling in CD4 T cells occurs through STAT3 and increased phosphorylated STAT3 can be found around bile ducts in PSC livers compared with controls.^86^ However, while JAK1/2 inhibitors experimentally blocked STAT3 and diminished the elevated IFN-γ responses, no effect on IL-17 was seen.^86^ Reduced and dysfunctional Tregs in PSC might be one factor allowing for elevated proinflammatory signaling. In more detail, the risk SNP in the *IL2RA* gene is associated with reduced circulating Tregs in PSC. One mechanism could be “less stable” Tregs because of weaker IL-2 signaling, reduced forkhead Box P3 (FoxP3) induction, and a lower level of FoxP3 demethylation.^87,88^ Similar results have been obtained in Mdr2^−/−^ mice with ensuing increased proinflammatory signals.^89^

What is then the origin of the pathogenic Th17 cells and where are they primed? Early work suggested gut priming, possibly then connected to dysbiosis, followed by liver-homing of T cells.^90^ However, more recent studies have shown that such a homing mechanism might not be specific to PSC.^91^ Nevertheless, TRMs in the bile ducts of patients with PSC express a combination of gut and liver-homing chemokine receptors.^9^ Break of tolerance in Mdr2^−/−^ mice by genetic removal of the inhibitory ectoenzyme CD39 promoted influx of T cells expressing the gut-homing integrin α4β7 to the liver.^92^ Intriguingly, a proportion of T cells in the gut and liver also share the same T-cell receptor clonotypes suggestive of a common origin.^93^

Altogether, this suggests that the balance between tolerance and reactivity with respect to T-cell responses is offset in PSC, leading to exaggerated Th17 and Th1 responses. However, most work until today has been performed in end-stage disease, not fully taking the distinct niches exiting within the liver into account.

### B cells

Compared with T cells, fewer studies have focused on the possible pathogenic role of B cells, despite the clinical overlap between PSC and many autoimmune disorders. Nevertheless, work deconvoluting B cells from bulk RNAseq revealed an increased B-cell signature in advanced PSC livers.^40^ These B cells could, in ST analysis of cirrhotic PSC livers, be localized to fibrotic regions as opposed to the parenchyma.^11^ Furthermore, a similar clonal overlap as has been described for T cells is also evident for B cells in the gut and liver.^94^ These clones had relatively shorter CDR3 lengths and a high degree of somatic hypermutations as compared with non-overlapping clonotypes, suggesting antigen-driven activation.^94^ Similar findings have been reported in another study focusing on the intestinal mucosa where PSC colitis was characterized by an increased proportion of IgG-secreting plasma cells, not seen to the same degree in non-PSC-IBD, where both IgG and IgA clones showed signs of affinity maturation.^95^ In Mdr2^−/−^ mice, circulating IgG and hepatic B cells increased upon fibrosis progression, and these hepatic B cells produced IgG in the absence of any stimulation.^96^ Moreover, in these mice, a much higher number of hepatic antibody-secreting cells was observed as compared with wild type, and this was associated with fibrosis progression.^96^ Dominant clonotypes were uniquely present in intrahepatic B cells from fibrotic as compared with nonfibrotic areas of the liver or splenic compartments, and analysis of somatic hypermutation rates in hepatic B cells suggested a liver-specific selection of the BCR repertoire occurring during hepatic fibrosis.^96^ Finally, Mdr2^−/−^ mice also had aberrant antibody concentration, elevated autoantibodies (ANA), and immune complexes, mimicking some of the features of patients with PSC. The depletion of B cells by anti-CD20 strongly reduced fibrosis, levels of IgG, ANA, immune complexes, and hepatic immune infiltrate,^96^ a therapeutic strategy that might have some bearing in humans.^97^ Furthermore, in vitro cultures of PSC explants also identified antibody-secreting cells secreting autoantibodies, and flow cytometry analysis revealed increased plasmablasts but fewer plasma cells as compared with primary biliary cholangitis.^98–100^ Finally, IgG4+ lymphoplasmacytic infiltration has been reported in PSC and seems to be associated with the presence of more severe disease.^101^ Indeed, more than 20% of explanted PSC livers contain increased IgG4+ periductal plasma cells.^102^ This is coupled with increased levels of antibodies against food and animal antigens in patients with PSC with high IgG4 levels, similar to IgG4-related disease.^103^ Taken together, although B-cell dysregulation is evident in PSC and elevated IgG in the blood commonly is seen in patients, mechanistic insights into such dysregulation remain to be elucidated. Furthermore, we are still lacking knowledge of the possible functional pathogenic roles of autoantibodies.

## CROSSTALK BETWEEN PARENCHYMA AND IMMUNE CELLS

While immune cells certainly contribute to PSC immunopathology, hepatocytes and cholangiocytes undergo phenotypic and functional changes in PSC that likely drive biliary inflammation and re-structuring and, therefore, disease trajectories. In addition to essential physiological functions, hepatocytes and cholangiocytes can sense pathogens through pattern recognition receptors and secrete antimicrobial peptides and inflammatory cytokines to initiate immunologic defenses.^104,105^ These immune functions are, therefore, critical for containing and controlling infection in the liver but may also contribute to sustained biliary damage upon persistent and chronic inflammation.^104^ Hepatocytes and cholangiocytes can interact with immune cells directly via antigen presentation and indirectly through cytokine secretion. Hepatocytes and cholangiocytes can express MHC class I molecules and are therefore capable of antigen presentation to CD8+ TRMs, the most abundant lymphocyte subset in human bile ducts.^9,106,107^ In mouse models, activated hepatocytes are capable of MHC class II upregulation for antigen presentation to CD4+ TRMs.^108^ MHC class II expression by cholangiocytes has also been observed in patients with PSC.^109^ As previously discussed, bile from PSC livers contains MAIT and NKT cell antigens, and cholangiocytes have been shown to present antigens to NKT cells through CD1d and to MAIT cells through MR1.^71,74,107^ Nevertheless, the exact role of hepatocyte and cholangiocyte antigen presentation in coordinating immune responses through classical and nonclassical MHC molecules remains unclear in humans and requires further study. As we understand today, other cell types, such as KCs and DCs, represent the professional antigen-presenting cells that initiate adaptive immune responses in the liver and biliary tract.^106^

Single-cell and spatial technologies have, in recent years, shed light upon nonimmune and immune phenotypes and interactions in PSC. Notably, a single-cell atlas has been generated using technologies including scRNAseq, single nuclear RNA sequencing, and ST on cirrhotic PSC livers to uncover novel cellular subsets associated with inflammation.^7^ One major finding was that CK7+HNF4A+ cholangiocyte-like hepatocytes were enriched in PSC livers relative to control livers.^7^ These transdifferentiating hepatocytes were marked by high levels of TNF and IFN-γ signaling, emphasizing their role in orchestrating immune-mediated inflammation in PSC.^7^ Additional novel findings include endothelial-mediated recruitment of monocyte-derived macrophages, which displayed an immunosuppressive phenotype in contrast to recruited lymphoid subsets in PSC livers.^7^ This research highlights the immunological complexity of PSC and provides a key data set for researchers to explore specific pathways of inflammation in PSC in more detail.

In recent years, cholangiocyte organoid models have been employed to study cholangiocyte-immune interactions ex vivo. scRNAseq of extrahepatic cholangiocyte organoids identified 8 cholangiocyte clusters ex vivo, including an inflammatory cluster marked by the expression of chemokines and TNF superfamily proteins.^21^ Of note, all 8 of these clusters were shared between PSC and non–PSC-derived organoids.^21^ However, PSC-derived cholangiocyte organoids were marked by an increased number of differentially expressed genes in response to IL-17 treatment relative to non–PSC-derived cholangiocyte organoids.^21^ In addition, supernatants from PSC cholangiocyte organoids were enriched for inflammatory proteins such as IL-6 and CXCL9, demonstrating their potential inflammatory phenotype in vivo.^21^ Beyond phenotypic assessment of cholangiocyte organoids ex vivo, cholangiocyte organoid-immune cell cocultures have been used to uncover potential pathogenic interactions between these cell types driving inflammation in PSC.^20^ Specifically, cholangiocytes were shown to mediate cleavage of CD100 expression on T cells, which resulted in a phenotypic skewing of these resident T cells toward a Th17 phenotype.^20^ Taken together, cholangiocyte organoids—and systems leveraging cholangiocyte-immune cell coculture—hold promise for discovering how interactions between cholangiocytes and immune cells propagate biliary inflammation and fibrosis in PSC.

Immune cells also have important effector functions targeting cholangiocytes. scRNAseq of PSC liver explants and Mdr2^−/−^ mice uncovered the role of CD8+ T cell and NK-cell–derived granzyme B (cytotoxic effector molecule) and TRAIL (apoptosis-inducing molecule) in PSC immunopathogenesis, both of which were elevated in PSC livers.^83^ Mdr2^−/−^ mice genetically lacking granzyme B demonstrated reduced cholangiocyte apoptosis, fibrosis, and liver injury relative to Mdr2^−/−^ control mice.^83^ Conversely, Mdr2^−/−^ mice genetically lacking TRAIL displayed elevated IFN-γ and granzyme B expression alongside elevated ALT levels and cholangiocyte apoptosis relative to Mdr2^−/−^ control mice.^83^ Further research into how inflammatory cytokines from recruited or resident immune cells modify cholangiocytes is therefore warranted.

Bi-directional cholangiocyte-immune cell crosstalk is, therefore, an important feature of PSC immunopathogenesis. Inflammatory cues from both cholangiocytes and immune cells likely contribute to a positive feedback loop of inflammation. Interrupting the cascade of inflammatory signaling at this axis holds promise in ameliorating structuring, fibrosis, and liver failure in PSC. Future studies should, therefore, explore this axis further. More sophisticated methods, such as “organ-on-a-chip” models incorporating multiple immune cell types and other supportive parenchymal cells, hold promise in elucidating these interactions between immune and nonimmune cells in more detail.

## IMPACT OF DYSBIOSIS, A LEAKY GUT, AND INTESTINAL INFLAMMATION ON BILIARY IMMUNE CELLS

The intricated relationship between PSC and IBD is difficult to dissect. Current studies estimate that 2%–7% of patients with IBD could have a concomitant PSC, often diagnosed later in life. In addition, 60%–90% of patients with PSC have IBD with specific features and represent a relatively milder progressive phenotype.^110,111^ In this context, the relationship between the gut and the liver, including its immunobiology, has been studied, mainly focusing on the intestinal microbiota, the mucosal inflammation, the associated leaky gut, and bile acid homeostasis. In this context, the shared clonal overlap of both T cells and B cells (both discussed above) between gut and liver is intriguing.^93,94^

Intestinal inflammation and associated gut dysbiosis are thought to disrupt bile acid homeostasis,^112,113^ a feature described in patients with PSC.^114,115^ Moreover, these bile acids have been shown to modulate the balance of Th17 cells and Tregs in experimental models.^112,116^ Recently, right-sided colonic biopsies from patients with PSC-IBD and IBD analyzed by bulk RNAseq, scRNAseq, and flow cytometry identified distinct features of intestinal inflammation in PSC-IBD compared with IBD where PSC-IBD inflammation was characterized by the presence of IL-17+FoxP3+ CD4 T cells as well as IgG-secreting plasma cells.^95^ In addition, even in the absence of IBD, an increased expression of *IL17A* and *IFNG* in intestinal biopsies and a dysbiotic microbiota was reported in the vast majority of patients with PSC.^117^ However, a study analyzing biliary brush samples from patients with PSC revealed no difference in biliary neutrophil or T-cell abundance in relation to IBD status.^9^ Gut inflammation increased mucosal permeability and translocation of bacterial product could potentially cause biliary inflammation. In such a context, an enhanced response of cholangiocytes to endotoxins has been reported in patients with PSC.^118^ In addition, human cholangiocytes exposed to bile from patients with PSC, which often contained microbial DNA, were able to in some cases activate MAIT cells in an MR1-dependent manner.^71^

PSC microbiota studies have revealed a global decrease in diversity associated with an overrepresentation of specific strains^119–122^ and some functional specificities.^123^ Results from germ-free mouse models suggested both protective and aggravated effects on cholestasis progression due to the microbiota.^124,125^ However, fecal microbiota from patients with PSC-IBD transferred to gnotobiotic mice led to, in some cases, activation of Th17 cells in the liver.^126^ These mice had increased susceptibility to DDC diet–induced hepatobiliary injury with higher serum bilirubin, ALP, and Th17 response in the liver as compared with germ-free mice.^126^ 16s rRNA sequencing of mesenteric lymph nodes identified *Klebsiella pneumoniae*, *Proteus mirabilis*, and *Enterococcus gallinarum* in those mice as well as in metagenomic analysis of PSC patient microbiota as compared with controls.^126^ In DDC-fed gnotobiotic mice colonized with these bacteria, inhibition of Th17 differentiation ameliorated hepatobiliary injury and fibrosis.^126^ Interestingly, *K. pneumoniae* derived from mesenteric lymph nodes was found to induce pores in colonic epithelial cells in vitro, and the use of a *K. pneumoniae* non–pore-forming strain in the 3-bacterial mix inoculated into germ-free mice led to decreased Th17 response in the liver.^126^

Although most patients with PSC also have IBD, the shared and specific immunobiological features of these entities remain poorly characterized. In recent years, studies have provided data on the microbiota composition of patients with PSC as well as their inflammatory landscape of the gut. Still, the mechanistic involvement of the liver and gut immune systems in this context remains poorly understood.

## IMMUNE-FOCUSED THERAPIES IN PSC—PAST, PRESENT, AND FUTURE

To date, no medical therapy has proven to have a significant impact on clinical outcomes in PSC.^1^ There are critical obstacles to the development of new therapies, such as a lack of statistical power and the slow and variable progression of the disease. Clinical trials are also hindered by the lack of disease activity markers associated with prognosis that can be used as endpoints. The window of treatment opportunity in PSC is unclear, and the point at which medical treatment may no longer be beneficial has not been identified.^127^ ALP, along with fibrosis progression markers (histology, liver stiffness measurement, and serum fibrosis markers), is the current gold standard endpoint but is insufficient due to a silent inflammatory process leading to disease progression that is difficult to detect.^128^ In addition, the patchy distribution of fibrosis makes histology and liver stiffness measurement variable and unreliable. Early-stage disease with low ALP levels, where immune-focused therapies are most likely to play a role, progresses very slowly.^129,130^ Consequently, drugs tested in short-term trials always risk being shown as ineffective, and patients with early-stage PSC with normal ALP levels are excluded from clinical trials. Conducting small, randomized studies over a short exposure time may result in potentially important drugs being dismissed despite their effectiveness.

The first randomized study in PSC was published in 1988, using D-penicillamine which was known to be effective in rheumatoid arthritis.^131^ Studies on corticosteroids followed, both orally and intrabiliary, and they showed no convincing beneficial effects but led to adverse metabolic and systemic effects.^132–137^ Therefore, the use of steroids is not recommended in the recent clinical guidelines to treat PSC.^138,139^ Very limited data exist on other immunosuppressive drugs. There are small, nonrandomized, trials or pilot studies of azathioprine,^136^ methotrexate,^140^ cyclosporine,^141^ tacrolimus,^142^ mycophenolate mofetil,^143^ and etanercept^144^ usage. While tacrolimus, mycophenolate mofetil, and cyclosporine showed modest biochemical improvements, these drugs demonstrated no significant treatment benefits to justify their use, especially given their substantial adverse effect profiles.^141^

The most used drug today is UDCA.^139,145^ UDCA improves ALP levels, but no clear survival benefit has been shown.^146^ Its mechanisms of action on the immune system are somewhat unclear, but UDCA is known to exert a direct effect on adaptive immunity by inhibiting DCs.^147^ The development of new immune therapies or biologics in IBD is rapidly advancing. Targeting gut inflammation through drugs or colectomy or the gut microbiome itself is hypothesized to play an important role in PSC progression as well. Unfortunately, immunotherapies effective for IBD have not demonstrated clear efficacy for PSC. Neither anti-TNF therapies (infliximab and adalimumab)^148–150^ nor vedolizumab (integrin α_4_β_7_ blockade)^151–153^ have been associated with improvements in cholestatic markers despite their effectiveness in treating IBD.^154^ Results from a retrospective study on the effect of the JAK inhibitor tofacitinib showed some promising results with improvement of cholestasis in PSC-IBD.^155^ There is one interesting ongoing phase II trial using CM-101 that targets the soluble protein CCL24, promoting fibrotic and inflammatory activities in the liver through its receptor, CCR3 (ClinicalTrials ID: NCT04595825). Finally, more experimental approaches, such as autologous hematopoietic stem cell transplantation^156^ and autologous Treg transfer^157^, have also been proposed.

Effective treatment is urgently needed for PSC. There are ongoing trials, including some experimental efforts. So far, immune-focused treatments, unfortunately have failed, but the rapidly growing field in IBD is also promising for PSC. However, further understanding of the immunopathology of PSC is required to choose the most promising immune treatment targets.

## SUMMARY AND OUTLOOK—SPACE AND TIME OF PSC

**FIGURE 2:**
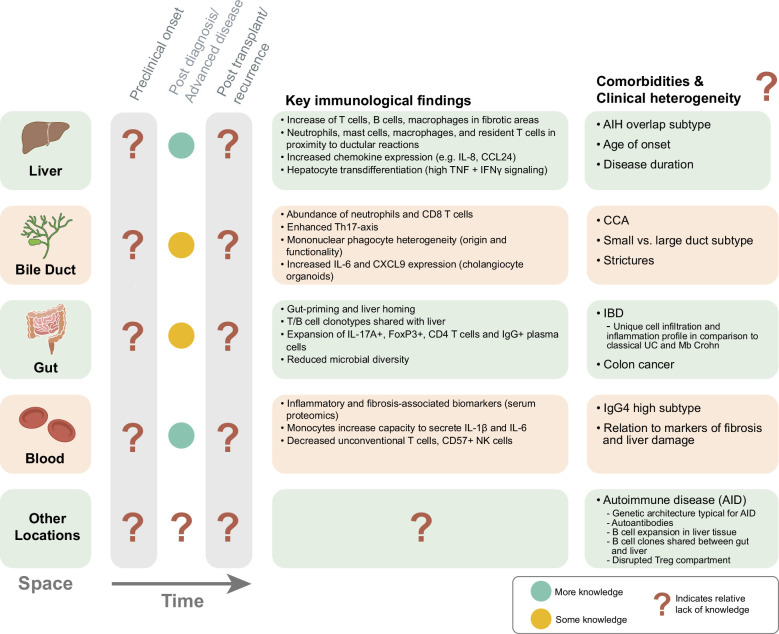
PSC pathogenesis through time, space, comorbidities, and clinical heterogeneity. Locations (space) of the immune studies are indicated in the left part of the figure. The extent of immunological knowledge in relation to disease stage (time) is thereafter depicted (preclinical, postdiagnosis/advanced disease, and posttransplant recurrent PSC). Most of the current immunobiological knowledge originates from a narrow spatiotemporal window, mostly from liver tissue postdiagnosis or in end-stage disease. The main immune features per location are detailed in bullet points. On the right side, related comorbidities and clinical heterogeneity–related aspects for each location are brought up. In most instances, it remains unclear how these aspects affect immunology. Abbreviation: PSC, primary sclerosing cholangitis.

We propose that the current immunobiological knowledge of PSC, to a greater extent, is placed into the context of time, space, and clinical presentation of the disease for improved interpretation (Figure 2). The time of study/sampling regarding the clinical course of the disease is an important aspect as PSC progression is observed over the course of several decades and can lead to a variable degree of fibrosis and multifocal biliary strictures. To date, most of the immunobiological knowledge, out of necessity, is from the time window of “post-diagnosis” to “end-stage disease” (Figure 2). Studying preclinical onset as well as posttransplant recurrent disease will likely provide additional key insights to better understand disease etiology and progression. While posttransplant recurrent disease will be easier to target in translational study designs, identifying cohorts of patients before the disease presents poses a challenge. However, clever usage of historical biobanks (serum and biopsies), patients with IBD who later present with PSC-IBD, as well as ongoing (or future) large population-based studies (UK Biobank and similar efforts), should likely yield translational opportunities, especially since novel technologies (proteomics, scRNAseq, and spatial methods) now permit detailed and broad analysis on for instance formal-fixed tissue.

Furthermore, although PSC is mostly studied and managed as a single disease entity, its clinical presentation and progression are heterogeneous, which should be looked upon when placing immunobiological knowledge into context. Indeed, PSC can affect both sexes, and the age at disease onset can span from childhood to late adulthood. These aspects might be of importance as both age and sex are associated with differences in the composition and function of the immune system.^158,159^ To date, limited immunological knowledge is available on these aspects to understand the predominance of PSC diagnosis in males in their 30s.

Regarding the spatial component of the disease, a large proportion of patients also have IBD with specific characteristics, as well as a high risk of developing cancer both in the biliary tract and the colon. As we know it today, the composition and function of the immune system vary in each tissue and evolve with age.^160^ Knowledge provided from the liver and the gut has revealed complementary immunological views of PSC. This highlights the need for looking at various tissue niches to better understand the complex interplay between tissues driving PSC pathogenesis. Beyond space at the organ level, technological development now also allows for spatial assessment of RNA, proteins, and metabolites at the single-cell level in situ.^5^ As discussed above, such efforts have already yielded novel insights into PSC pathogenesis, and it is clear that the biliary niche is distinct from that of the liver parenchyma.^5,9^ Future work will likely benefit from an even greater consideration of events occurring within micro-niches.

Finally, the heterogeneity of PSC is driven by additional features such as the levels of severity of disease, secondary effects of the presence of high-grade strictures, recurrent bacterial cholangitis, circulating IgG4, the involvement of small or large bile duct, or the presence of autoimmune hepatitis features. Immunological investigations are needed to better dissect these PSC subtypes. Specifically, as the portfolio of immunotherapeutic agents is expanding, immunobiological knowledge placed in the context of time, space, and heterogeneity of PSC might provide future options for precision medicine.

## References

[1] Karlsen TH Folseraas T Thorburn D Vesterhus M. Primary sclerosing cholangitis—A comprehensive review. J Hepatol. 2017;67:1298–1323. 28802875 10.1016/j.jhep.2017.07.022

[2] Ricciuto A Kamath BM Hirschfield GM Trivedi PJ. Primary sclerosing cholangitis and overlap features of autoimmune hepatitis: A coming of age or an age-ist problem? J Hepatol. 2023;79:567–575. 36870613 10.1016/j.jhep.2023.02.030

[3] Jiang X Karlsen TH. Genetics of primary sclerosing cholangitis and pathophysiological implications. Nat Rev Gastroenterol Hepatol. 2017;14:279–295. 28293027 10.1038/nrgastro.2016.154

[4] Sebode M Weiler-Normann C Liwinski T Schramm C. Autoantibodies in autoimmune liver disease—Clinical and diagnostic relevance. Front Immunol. 2018;9:609. 29636752 10.3389/fimmu.2018.00609 PMC5880919

[5] Björkström NK. Immunobiology of the biliary tract system. J Hepatol. 2022;77:1657–1669. 36116989 10.1016/j.jhep.2022.08.018 PMC7615184

[6] Björkström NK Ljunggren HG Michaëlsson J. Emerging insights into natural killer cells in human peripheral tissues. Nat Rev Immunol. 2016;16:310–320. 27121652 10.1038/nri.2016.34

[7] Andrews TS Nakib D Perciani CT Ma XZ Liu L Winter E. Single-cell, single-nucleus, and spatial transcriptomics characterization of the immunological landscape in the healthy and PSC human liver. J Hepatol. 2024;80:730–743. 38199298 10.1016/j.jhep.2023.12.023

[8] Poch T Krause J Casar C Liwinski T Glau L Kaufmann M. Single-cell atlas of hepatic T cells reveals expansion of liver-resident naive-like CD4(+) T cells in primary sclerosing cholangitis. J Hepatol. 2021;75:414–423. 33774059 10.1016/j.jhep.2021.03.016 PMC8310924

[9] Zimmer CL von Seth E Buggert M Strauss O Hertwig L Nguyen S. A biliary immune landscape map of primary sclerosing cholangitis reveals a dominant network of neutrophils and tissue-resident T cells. Sci Transl Med. 2021;13:eabb3107. 34162753 10.1126/scitranslmed.abb3107

[10] Guilliams M Bonnardel J Haest B Vanderborght B Wagner C Remmerie A. Spatial proteogenomics reveals distinct and evolutionarily conserved hepatic macrophage niches. Cell. 2022;185:379–396. 35021063 10.1016/j.cell.2021.12.018 PMC8809252

[11] Chung BK Øgaard J Reims HM Karlsen TH Melum E. Spatial transcriptomics identifies enriched gene expression and cell types in human liver fibrosis. Hepatol Commun. 2022;6:2538–2550. 35726350 10.1002/hep4.2001 PMC9426406

[12] Shafqat A Khan JA Alkachem AY Sabur H Alkattan K Yaqinuddin A. How neutrophils shape the immune response: Reassessing their multifaceted role in health and disease. Int J Mol Sci. 2023;24:17583. 38139412 10.3390/ijms242417583 PMC10744338

[13] Herrero-Cervera A Soehnlein O Kenne E. Neutrophils in chronic inflammatory diseases. Cell Mol Immunol. 2022;19:177–191. 35039631 10.1038/s41423-021-00832-3 PMC8803838

[14] Luci C Bourinet M Leclère PS Anty R Gual P. Chronic inflammation in non-alcoholic steatohepatitis: Molecular mechanisms and therapeutic strategies. Front Endocrinol (Lausanne). 2020;11:597648. 33384662 10.3389/fendo.2020.597648 PMC7771356

[15] Hintermann E Tondello C Fuchs S Bayer M Pfeilschifter JM Taubert R. Blockade of neutrophil extracellular trap components ameliorates cholestatic liver disease in Mdr2 (Abcb4) knockout mice. J Autoimmun. 2024;146:103229. 38653165 10.1016/j.jaut.2024.103229

[16] Greenman R Segal-Salto M Barashi N Hay O Katav A Levi O. CCL24 regulates biliary inflammation and fibrosis in primary sclerosing cholangitis. JCI Insight. 2023;8:e162270. 37345655 10.1172/jci.insight.162270 PMC10371243

[17] Greenman R Snir T Katav A Aricha R Mishalian I Hay O. The role of CCL24 in primary sclerosing cholangitis: Bridging patient serum proteomics to preclinical data. Cells. 2024;13:209. 38334601 10.3390/cells13030209 PMC10854794

[18] Zweers SJ Shiryaev A Komuta M Vesterhus M Hov JR Perugorria MJ. Elevated interleukin-8 in bile of patients with primary sclerosing cholangitis. Liver Int. 2016;36:1370–1377. 26866350 10.1111/liv.13092

[19] Mor A Friedman S Hashmueli S Peled A Pinzani M Frankel M. Targeting CCL24 in inflammatory and fibrotic diseases: Rationale and results from three CM-101 phase 1 studies. Drug Saf. 2024;47:869–881. 38822943 10.1007/s40264-024-01436-2 PMC11324678

[20] Jiang X Otterdal K Chung BK Maucourant C Rønneberg JD Zimmer CL. Cholangiocytes modulate CD100 expression in the liver and facilitate pathogenic T-helper 17 cell differentiation. Gastroenterology. 2024;166:667–679. 37995866 10.1053/j.gastro.2023.11.283

[21] Garcia Moreno AS Guicciardi ME Wixom AQ Jessen E Yang J Ilyas SI. IL-17 signaling in primary sclerosing cholangitis patient-derived organoids. Hepatol Commun. 2024;8:e0454. 38829197 10.1097/HC9.0000000000000454 PMC11150034

[22] Schön MP Erpenbeck L. The interleukin-23/interleukin-17 axis links adaptive and innate immunity in psoriasis. Front Immunol. 2018;9:1323. 29963046 10.3389/fimmu.2018.01323 PMC6013559

[23] Kvedaraite E. Neutrophil-T cell crosstalk in inflammatory bowel disease. Immunology. 2021;164:657–664. 34240423 10.1111/imm.13391 PMC8561100

[24] Iliopoulou L Lianopoulou E Kollias G. IL-23 exerts dominant pathogenic functions in Crohn's disease-ileitis. Mucosal Immunol. 2024. S1933-0219(24)00049-7. doi: 10.1016/j.mucimm.2024.05.008 38844209

[25] Griffin GK Newton G Tarrio ML Bu DX Maganto-Garcia E Azcutia V. IL-17 and TNF-α sustain neutrophil recruitment during inflammation through synergistic effects on endothelial activation. J Immunol. 2012;188:6287–6299. 22566565 10.4049/jimmunol.1200385 PMC3370121

[26] Lourda M Dzidic M Hertwig L Bergsten H Palma Medina LM Sinha I. High-dimensional profiling reveals phenotypic heterogeneity and disease-specific alterations of granulocytes in COVID-19. Proc Natl Acad Sci USA. 2021;118:e2109123118. 34548411 10.1073/pnas.2109123118 PMC8501786

[27] Townsend L Dyer AH Naughton A Imangaliyev S Dunne J Kiersey R. Severe COVID-19 is characterised by inflammation and immature myeloid cells early in disease progression. Heliyon. 2022;8:09230. 10.1016/j.heliyon.2022.e09230 PMC8973020 35386227

[28] Jaillon S Ponzetta A Di Mitri D Santoni A Bonecchi R Mantovani A. Neutrophil diversity and plasticity in tumour progression and therapy. Nat Rev Cancer. 2020;20:485–503. 32694624 10.1038/s41568-020-0281-y

[29] Coudereau R Waeckel L Cour M Rimmele T Pescarmona R Fabri A. Emergence of immunosuppressive LOX-1+ PMN-MDSC in septic shock and severe COVID-19 patients with acute respiratory distress syndrome. J Leukoc Biol. 2022;111:489–496. 33909917 10.1002/JLB.4COVBCR0321-129R PMC8242532

[30] Uhl B Vadlau Y Zuchtriegel G Nekolla K Sharaf K Gaertner F. Aged neutrophils contribute to the first line of defense in the acute inflammatory response. Blood. 2016;128:2327–2337. 27609642 10.1182/blood-2016-05-718999 PMC5122310

[31] Ariño S Aguilar-Bravo B Coll M Lee WY Peiseler M Cantallops-Vilà P. Ductular reaction-associated neutrophils promote biliary epithelium proliferation in chronic liver disease. J Hepatol. 2023;79:1025–1036. 37348790 10.1016/j.jhep.2023.05.045 PMC10585421

[32] Zwicker C Bujko A Scott CL. Hepatic macrophage responses in inflammation, a function of plasticity, heterogeneity or both? Front Immunol. 2021;12:690813. 34177948 10.3389/fimmu.2021.690813 PMC8220199

[33] Kunzmann LK Schoknecht T Poch T Henze L Stein S Kriz M. Monocytes as potential mediators of pathogen-induced T-helper 17 differentiation in patients with primary sclerosing cholangitis (PSC). Hepatology. 2020;72:1310–1326. 33090557 10.1002/hep.31140

[34] Barreby E Strunz B Nock S Naudet L Shen JX Johansson H. Human resident liver myeloid cells protect against metabolic stress in obesity. Nat Metab. 2023;5:1188–1203. 37414931 10.1038/s42255-023-00834-7 PMC10365994

[35] Chen YY Arndtz K Webb G Corrigan M Akiror S Liaskou E. Intrahepatic macrophage populations in the pathophysiology of primary sclerosing cholangitis. JHEP Rep. 2019;1:369–376. 32039388 10.1016/j.jhepr.2019.10.003 PMC7005651

[36] Stankey CT Bourges C Haag LM Turner-Stokes T Piedade AP Palmer-Jones C. A disease-associated gene desert directs macrophage inflammation through ETS2. Nature. 2024;630:447–456. 38839969 10.1038/s41586-024-07501-1 PMC11168933

[37] Guicciardi ME Trussoni CE Krishnan A Bronk SF Lorenzo Pisarello MJ O'hara SP. Macrophages contribute to the pathogenesis of sclerosing cholangitis in mice. J Hepatol. 2018;69:676–686. 29802947 10.1016/j.jhep.2018.05.018 PMC6098983

[38] Miyamoto Y Kikuta J Matsui T Hasegawa T Fujii K Okuzaki D. Periportal macrophages protect against commensal-driven liver inflammation. Nature. 2024;629:901–909. 38658756 10.1038/s41586-024-07372-6

[39] Govaere O Cockell S Van Haele M Wouters J Van Delm W Van den Eynde K. High-throughput sequencing identifies aetiology-dependent differences in ductular reaction in human chronic liver disease. J Pathol. 2019;248:66–76. 30584802 10.1002/path.5228

[40] Guillot A Winkler M Silva Afonso M Aggarwal A Lopez D Berger H. Mapping the hepatic immune landscape identifies monocytic macrophages as key drivers of steatohepatitis and cholangiopathy progression. Hepatology. 2023;78:150–166. 36630995 10.1097/HEP.0000000000000270

[41] Labiano I Agirre-Lizaso A Olaizola P Echebarria A Huici-Izagirre M Olaizola I. TREM-2 plays a protective role in cholestasis by acting as a negative regulator of inflammation. J Hepatol. 2022;77:991–1004. 35750136 10.1016/j.jhep.2022.05.044

[42] De Muynck K Heyerick L De Ponti FF Vanderborght B Meese T Van Campenhout S. Osteopontin characterizes bile duct-associated macrophages and correlates with liver fibrosis severity in primary sclerosing cholangitis. Hepatology. 2024;79:269–288. 37535809 10.1097/HEP.0000000000000557 PMC10789378

[43] Chen R Huang B Lian M Wei Y Miao Q Liang J. A+T rich interaction domain protein 3a (Arid3a) impairs Mertk-mediated efferocytosis in cholestasis. J Hepatol. 2023;79:1478–1490. 37659731 10.1016/j.jhep.2023.08.016

[44] De Muynck K Vanderborght B De Ponti FF Gijbels E Van Welden S Guilliams M. Kupffer cells contested as early drivers in the pathogenesis of primary sclerosing cholangitis. Am J Pathol. 2023;193:366–379. 36642171 10.1016/j.ajpath.2022.12.008

[45] Kvedaraite E Ginhoux F. Human dendritic cells in cancer. Sci Immunol. 2022;7:9409. 10.1126/sciimmunol.abm9409 35363544

[46] Heras-Murillo I Adán-Barrientos I Galán M Wculek SK Sancho D. Dendritic cells as orchestrators of anticancer immunity and immunotherapy. Nat Rev Clin Oncol. 2024;21:257–277. 38326563 10.1038/s41571-024-00859-1

[47] Müller AL Casar C Preti M Krzikalla D Gottwick C Averhoff P. Inflammatory type 2 conventional dendritic cells contribute to murine and human cholangitis. J Hepatol. 2022;77:1532–1544. 35798133 10.1016/j.jhep.2022.06.025

[48] Weiskirchen R Meurer SK Liedtke C Huber M. Mast cells in liver fibrogenesis. Cells. 2019;8:1429. 31766207 10.3390/cells8111429 PMC6912398

[49] Huang S Wu H Luo F Zhang B Li T Yang Z. Exploring the role of mast cells in the progression of liver disease. Front Physiol. 2022;13:964887. 36176778 10.3389/fphys.2022.964887 PMC9513450

[50] Meadows V Kennedy L Ekser B Kyritsi K Kundu D Zhou T. Mast cells regulate ductular reaction and intestinal inflammation in cholestasis through farnesoid X receptor signaling. Hepatology. 2021;74:2684–2698. 34164827 10.1002/hep.32028 PMC9337218

[51] Ishii M Iwai M Harada Y Morikawa T Okanoue T Kishikawa T. A role of mast cells for hepatic fibrosis in primary sclerosing cholangitis. Hepatol Res. 2005;31:127–131. 15777700 10.1016/j.hepres.2005.01.007

[52] Jones H Hargrove L Kennedy L Meng F Graf-Eaton A Owens J. Inhibition of mast cell-secreted histamine decreases biliary proliferation and fibrosis in primary sclerosing cholangitis Mdr2(-/-) mice. Hepatology. 2016;64:1202–1216. 27351144 10.1002/hep.28704 PMC5033697

[53] Kennedy L Hargrove L Demieville J Karstens W Jones H DeMorrow S. Blocking H1/H2 histamine receptors inhibits damage/fibrosis in Mdr2(-/-) mice and human cholangiocarcinoma tumorigenesis. Hepatology. 2018;68:1042–1056. 29601088 10.1002/hep.29898 PMC6165706

[54] Meadows V Marakovits C Ekser B Kundu D Zhou T Kyritsi K. Loss of apical sodium bile acid transporter alters bile acid circulation and reduces biliary damage in cholangitis. Am J Physiol Gastrointest Liver Physiol. 2023;324:60. 10.1152/ajpgi.00112.2022 PMC9799145 36410025

[55] Meng F Kennedy L Hargrove L Demieville J Jones H Madeka T. Ursodeoxycholate inhibits mast cell activation and reverses biliary injury and fibrosis in Mdr2(-/-) mice and human primary sclerosing cholangitis. Lab Invest. 2018;98:1465–1477. 30143751 10.1038/s41374-018-0101-0 PMC6214746

[56] Björkström NK Strunz B Ljunggren HG. Natural killer cells in antiviral immunity. Nat Rev Immunol. 2022;22:112–123. 34117484 10.1038/s41577-021-00558-3 PMC8194386

[57] Fuchs A Vermi W Lee JS Lonardi S Gilfillan S Newberry RD. Intraepithelial type 1 innate lymphoid cells are a unique subset of IL-12- and IL-15-responsive IFN-γ-producing cells. Immunity. 2013;38:769–781. 23453631 10.1016/j.immuni.2013.02.010 PMC3634355

[58] Nagasawa M Spits H Ros XR. Innate lymphoid cells (ILCs): Cytokine hubs regulating immunity and tissue homeostasis. Cold Spring Harb Perspect Biol. 2018;10:a030304. 29229782 10.1101/cshperspect.a030304 PMC6280706

[59] Yudanin NA Schmitz F Flamar AL Thome JJC Tait Wojno E Moeller JB. Spatial and temporal mapping of human innate lymphoid cells reveals elements of tissue specificity. Immunity. 2019;50:505–519. 30770247 10.1016/j.immuni.2019.01.012 PMC6594374

[60] Klose CS Artis D. Innate lymphoid cells as regulators of immunity, inflammation and tissue homeostasis. Nat Immunol. 2016;17:765–774. 27328006 10.1038/ni.3489

[61] Marquardt N Béziat V Nyström S Hengst J Ivarsson MA Kekäläinen E. Cutting edge: Identification and characterization of human intrahepatic CD49a+ NK cells. J Immunol. 2015;194:2467–2471. 25672754 10.4049/jimmunol.1402756

[62] Mikulak J Bruni E Oriolo F Di Vito C Mavilio D. Hepatic natural killer cells: Organ-specific sentinels of liver immune homeostasis and physiopathology. Front Immunol. 2019;10:946. 31114585 10.3389/fimmu.2019.00946 PMC6502999

[63] Cuff AO Robertson FP Stegmann KA Pallett LJ Maini MK Davidson BR. Eomeshi NK cells in human liver are long-lived and do not recirculate but can be replenished from the circulation. J Immunol. 2016;197:4283–4291. 27798170 10.4049/jimmunol.1601424 PMC5114885

[64] Liu B Yang GX Sun Y Tomiyama T Zhang W Leung PSC. Decreased CD57 expression of natural killer cells enhanced cytotoxicity in patients with primary sclerosing cholangitis. Front Immunol. 2022;13:912961. 36059513 10.3389/fimmu.2022.912961 PMC9434697

[65] Zecher BF Ellinghaus D Schloer S Niehrs A Padoan B Baumdick ME. HLA-DPA1*02:01~B1*01:01 is a risk haplotype for primary sclerosing cholangitis mediating activation of NKp44+ NK cells. Gut. 2024;73:325–337. 37788895 10.1136/gutjnl-2023-329524 PMC10850656

[66] Pisarska MM Dunne MR O’Shea D Hogan AE. Interleukin-17 producing mucosal associated invariant T cells—Emerging players in chronic inflammatory diseases? Eur J Immunol. 2020;50:1098–1108. 32617963 10.1002/eji.202048645

[67] Godfrey DI Uldrich AP McCluskey J Rossjohn J Moody DB. The burgeoning family of unconventional T cells. Nat Immunol. 2015;16:1114–1123. 26482978 10.1038/ni.3298

[68] Groh V Rhinehart R Secrist H Bauer S Grabstein KH Spies T. Broad tumor-associated expression and recognition by tumor-derived gamma delta T cells of MICA and MICB. Proc Natl Acad Sci USA. 1999;96:6879–6884. 10359807 10.1073/pnas.96.12.6879 PMC22010

[69] Hu Y Hu Q Li Y Lu L Xiang Z Yin Z. γδ T cells: Origin and fate, subsets, diseases and immunotherapy. Signal Transduct Target Ther. 2023;8:434. 37989744 10.1038/s41392-023-01653-8 PMC10663641

[70] von Seth E Zimmer CL Reuterwall-Hansson M Barakat A Arnelo U Bergquist A. Primary sclerosing cholangitis leads to dysfunction and loss of MAIT cells. Eur J Immunol. 2018;48:1997–2004. 30252934 10.1002/eji.201847608

[71] Valestrand L Zheng F Hansen SH Øgaard J Hov JR Björkström NK. Bile from patients with primary sclerosing cholangitis contains mucosal-associated invariant T-cell antigens. Am J Pathol. 2022;192:629–641. 35063408 10.1016/j.ajpath.2021.12.008 PMC12178327

[72] Ito E Inuki S Izumi Y Takahashi M Dambayashi Y Ciacchi L. Sulfated bile acid is a host-derived ligand for MAIT cells. Sci Immunol. 2024;9:6924. 10.1126/sciimmunol.ade6924 PMC11147531 38277465

[73] du Halgouet A Darbois A Alkobtawi M Mestdagh M Alphonse A Premel V. Role of MR1-driven signals and amphiregulin on the recruitment and repair function of MAIT cells during skin wound healing. Immunity. 2023;56:78–92. 36630919 10.1016/j.immuni.2022.12.004 PMC9839364

[74] Valestrand L Berntsen NL Zheng F Schrumpf E Hansen SH Karlsen TH. Lipid antigens in bile from patients with chronic liver diseases activate natural killer T cells. Clin Exp Immunol. 2021;203:304–314. 33089489 10.1111/cei.13541 PMC7806449

[75] Berntsen NL Fosby B Tan C Reims HM Ogaard J Jiang X. Natural killer T cells mediate inflammation in the bile ducts. Mucosal Immunol. 2018;11:1582–1590. 30115993 10.1038/s41385-018-0066-8 PMC6402771

[76] Tedesco D Thapa M Chin CY Ge Y Gong M Li J. Alterations in intestinal microbiota lead to production of interleukin 17 by intrahepatic γδ T-cell receptor-positive cells and pathogenesis of cholestatic liver disease. Gastroenterology. 2018;154:2178–2193. 29454797 10.1053/j.gastro.2018.02.019 PMC5985208

[77] Melum E Franke A Schramm C Weismüller TJ Gotthardt DN Offner FA. Genome-wide association analysis in primary sclerosing cholangitis identifies two non-HLA susceptibility loci. Nat Genet. 2011;43:17–19. 21151127 10.1038/ng.728 PMC4354850

[78] Liu JZ Hov JR Folseraas T Ellinghaus E Rushbrook SM Doncheva NT. Dense genotyping of immune-related disease regions identifies nine new risk loci for primary sclerosing cholangitis. Nat Genet. 2013;45:670–675. 23603763 10.1038/ng.2616 PMC3667736

[79] Pratt HE Wu T Elhajjajy S Zhou J Fitzgerald K Fazzio T. Beyond genome-wide association studies: Investigating the role of noncoding regulatory elements in primary sclerosing cholangitis. Hepatol Commun. 2023;7:e0242. 37756045 10.1097/HC9.0000000000000242 PMC10531193

[80] Jiang X Bergquist A Löscher BS Venkatesh G Mold JE Holm K. A heterozygous germline CD100 mutation in a family with primary sclerosing cholangitis. Sci Transl Med. 2021;13:eabb0036. 33627483 10.1126/scitranslmed.abb0036

[81] Li Y Li B Xiao X Qian Q Wang R Lyu Z. Itaconate inhibits CD103 + T RM cells and alleviates hepatobiliary injury in mouse models of primary sclerosing cholangitis. Hepatology. 2024;79:25–38. 37505225 10.1097/HEP.0000000000000549

[82] Poch T Bahn J Casar C Krause J Evangelakos I Gilladi H. Intergenic risk variant rs56258221 skews the fate of naive CD4(+) T cells via miR4464-BACH2 interplay in primary sclerosing cholangitis. Cell Rep Med. 2024;5:101620. 38901430 10.1016/j.xcrm.2024.101620 PMC11293351

[83] Kellerer M Javed S Casar C Will N Berkhout LK Schwinge D. Antagonistic effects of the cytotoxic molecules granzyme B and TRAIL in the immunopathogenesis of sclerosing cholangitis. Hepatology. 2024. Epub ahead of print. doi: 10.1097/HEP.0000000000000830 PMC11407778 38441998

[84] Ravichandran G Neumann K Berkhout LK Weidemann S Langeneckert AE Schwinge D. Interferon-γ-dependent immune responses contribute to the pathogenesis of sclerosing cholangitis in mice. J Hepatol. 2019;71:773–782. 31173810 10.1016/j.jhep.2019.05.023

[85] Reyes JL Vannan DT Vo T Gulamhusein A Beck PL Reimer RA. Neutralization of IL-15 abrogates experimental immune-mediated cholangitis in diet-induced obese mice. Sci Rep. 2018;8:3127. 29449577 10.1038/s41598-018-21112-7 PMC5814438

[86] Dold L Frank L Lutz P Kaczmarek DJ Krämer B Nattermann J. IL-6-dependent STAT3 activation and induction of proinflammatory cytokines in primary sclerosing cholangitis. Clin Transl Gastroenterol. 2023;14:00603. 10.14309/ctg.0000000000000603 PMC10461951 37256725

[87] Sebode M Peiseler M Franke B Schwinge D Schoknecht T Wortmann F. Reduced FOXP3(+) regulatory T cells in patients with primary sclerosing cholangitis are associated with IL2RA gene polymorphisms. J Hepatol. 2014;60:1010–1016. 24412607 10.1016/j.jhep.2013.12.027

[88] Dold L Kalthoff S Frank L Zhou T Esser P Lutz P. STAT activation in regulatory CD4(+) T cells of patients with primary sclerosing cholangitis. Immun Inflamm Dis. 2024;12:1248. 10.1002/iid3.1248 PMC11010953 38607233

[89] Schwinge D von Haxthausen F Quaas A Carambia A Otto B Glaser F. Dysfunction of hepatic regulatory T cells in experimental sclerosing cholangitis is related to IL-12 signaling. J Hepatol. 2017;66:798–805. 27965154 10.1016/j.jhep.2016.12.001

[90] Eksteen B Grant AJ Miles A Curbishley SM Lalor PF Hübscher SG. Hepatic endothelial CCL25 mediates the recruitment of CCR9+ gut-homing lymphocytes to the liver in primary sclerosing cholangitis. J Exp Med. 2004;200:1511–1517. 15557349 10.1084/jem.20041035 PMC2211943

[91] Graham JJ Mukherjee S Yuksel M Sanabria Mateos R Si T Huang Z. Aberrant hepatic trafficking of gut-derived T cells is not specific to primary sclerosing cholangitis. Hepatology. 2022;75:518–530. 34633679 10.1002/hep.32193 PMC8844147

[92] Peng ZW Rothweiler S Wei G Ikenaga N Liu SB Sverdlov DY. The ectonucleotidase ENTPD1/CD39 limits biliary injury and fibrosis in mouse models of sclerosing cholangitis. Hepatol Commun. 2017;1:957–972. 29404503 10.1002/hep4.1084 PMC5721459

[93] Henriksen EK Jørgensen KK Kaveh F Holm K Hamm D Olweus J. Gut and liver T-cells of common clonal origin in primary sclerosing cholangitis-inflammatory bowel disease. J Hepatol. 2017;66:116–122. 27647428 10.1016/j.jhep.2016.09.002

[94] Chung BK Henriksen EKK Jørgensen KK Karlsen TH Hirschfield GM Liaskou E. Gut and liver B cells of common clonal origin in primary sclerosing cholangitis-inflammatory bowel disease. Hepatol Commun. 2018;2:956–967. 30094406 10.1002/hep4.1200 PMC6078219

[95] Shaw DG Aguirre-Gamboa R Vieira MC Gona S DiNardi N Wang A. Antigen-driven colonic inflammation is associated with development of dysplasia in primary sclerosing cholangitis. Nat Med. 2023;29:1520–1529. 37322120 10.1038/s41591-023-02372-x PMC10287559

[96] Thapa M Tedesco D Gumber S Elrod EJ Han JH Kitchens WH. Blockade of BAFF reshapes the hepatic B cell receptor repertoire and attenuates autoantibody production in cholestatic liver disease. J Immunol. 2020;204:3117–3128. 32332110 10.4049/jimmunol.1900391 PMC7306885

[97] Yamada Y Hoshino K Fuchimoto Y Matsubara K Hibi T Yagi H. Rituximab induction to prevent the recurrence of PSC after liver transplantation—The lessons learned from ABO-incompatible living donor liver transplantation. Transplant Direct. 2018;4:342. 10.1097/TXD.0000000000000760 PMC5811271 29464203

[98] Chung BK Guevel BT Reynolds GM Gupta Udatha DB Henriksen EK Stamataki Z. Phenotyping and auto-antibody production by liver-infiltrating B cells in primary sclerosing cholangitis and primary biliary cholangitis. J Autoimmun. 2017;77:45–54. 27784538 10.1016/j.jaut.2016.10.003

[99] Zhang J Zhang W Leung PS Bowlus CL Dhaliwal S Coppel RL. Ongoing activation of autoantigen-specific B cells in primary biliary cirrhosis. Hepatology. 2014;60:1708–1716. 25043065 10.1002/hep.27313 PMC4211937

[100] Zen Y Quaglia A Portmann B. Immunoglobulin G4-positive plasma cell infiltration in explanted livers for primary sclerosing cholangitis. Histopathology. 2011;58:414–422. 21348891 10.1111/j.1365-2559.2011.03763.x

[101] Fischer S Trivedi PJ Ward S Greig PD Therapondos G Hirschfield GM. Frequency and significance of IgG4 immunohistochemical staining in liver explants from patients with primary sclerosing cholangitis. Int J Exp Pathol. 2014;95:209–215. 24750423 10.1111/iep.12076 PMC4351857

[102] Zhang L Lewis JT Abraham SC Smyrk TC Leung S Chari ST. IgG4+ plasma cell infiltrates in liver explants with primary sclerosing cholangitis. Am J Surg Pathol. 2010;34:88–94. 20035148 10.1097/PAS.0b013e3181c6c09a

[103] Culver EL Vermeulen E Makuch M van Leeuwen A Sadler R Cargill T. Increased IgG4 responses to multiple food and animal antigens indicate a polyclonal expansion and differentiation of pre-existing B cells in IgG4-related disease. Ann Rheum Dis. 2015;74:944–947. 25646372 10.1136/annrheumdis-2014-206405 PMC4392210

[104] Pinto C Giordano DM Maroni L Marzioni M. Role of inflammation and proinflammatory cytokines in cholangiocyte pathophysiology. Biochim Biophys Acta Mol Basis Dis. 2018;1864:1270–1278. 28754451 10.1016/j.bbadis.2017.07.024

[105] Huang C Zhou Y Cheng J Guo X Shou D Quan Y. Pattern recognition receptors in the development of nonalcoholic fatty liver disease and progression to hepatocellular carcinoma: An emerging therapeutic strategy. Front Endocrinol (Lausanne). 2023;14:1145392. 37020586 10.3389/fendo.2023.1145392 PMC10067914

[106] Grakoui A Crispe IN. Presentation of hepatocellular antigens. Cell Mol Immunol. 2016;13:293–300. 26924525 10.1038/cmi.2015.109 PMC4856799

[107] Schrumpf E Tan C Karlsen TH Sponheim J Björkström NK Sundnes O. The biliary epithelium presents antigens to and activates natural killer T cells. Hepatology. 2015;62:1249–1259. 25855031 10.1002/hep.27840 PMC4589438

[108] Herkel J Jagemann B Wiegard C Lazaro JF Lueth S Kanzler S. MHC class II-expressing hepatocytes function as antigen-presenting cells and activate specific CD4 T lymphocyutes. Hepatology. 2003;37:1079–1085. 12717388 10.1053/jhep.2003.50191

[109] Chapman RW Kelly PM Heryet A Jewell DP Fleming KA. Expression of HLA-DR antigens on bile duct epithelium in primary sclerosing cholangitis. Gut. 1988;29:422–427. 3286382 10.1136/gut.29.4.422 PMC1433533

[110] Kim YS Hurley EH Park Y Ko S. Primary sclerosing cholangitis (PSC) and inflammatory bowel disease (IBD): A condition exemplifying the crosstalk of the gut-liver axis. Exp Mol Med. 2023;55:1380–1387. 37464092 10.1038/s12276-023-01042-9 PMC10394020

[111] van Munster KN Bergquist A Ponsioen CY. Inflammatory bowel disease and primary sclerosing cholangitis: One disease or two? J Hepatol. 2024;80:155–168. 37940453 10.1016/j.jhep.2023.09.031

[112] Jia W Xie G Jia W. Bile acid-microbiota crosstalk in gastrointestinal inflammation and carcinogenesis. Nat Rev Gastroenterol Hepatol. 2018;15:111–128. 29018272 10.1038/nrgastro.2017.119 PMC5899973

[113] Sagar NM Cree IA Covington JA Arasaradnam RP. The interplay of the gut microbiome, bile acids, and volatile organic compounds. Gastroenterol Res Pract. 2015;2015:398585. 25821460 10.1155/2015/398585 PMC4363917

[114] Bell LN Wulff J Comerford M Vuppalanchi R Chalasani N. Serum metabolic signatures of primary biliary cirrhosis and primary sclerosing cholangitis. Liver Int. 2015;35:263–274. 25181933 10.1111/liv.12680 PMC4293304

[115] Quraishi MN Acharjee A Beggs AD Horniblow R Tselepis C Gkoutos G. A pilot integrative analysis of colonic gene expression, gut microbiota, and immune infiltration in primary sclerosing cholangitis-inflammatory bowel disease: Association of disease with bile acid pathways. J Crohns Colitis. 2020;14:935–947. 32016358 10.1093/ecco-jcc/jjaa021 PMC7392170

[116] Hang S Paik D Yao L Kim E Trinath J Lu J. Bile acid metabolites control T(H)17 and T(reg) cell differentiation. Nature. 2019;576:143–148. 31776512 10.1038/s41586-019-1785-z PMC6949019

[117] Wittek A Steglich B Casar C Seiz O Huber P Ehlken H. A gradient of intestinal inflammation in primary sclerosing cholangitis. Inflamm Bowel Dis. 2024;30:900–910. 37540889 10.1093/ibd/izad137

[118] Mueller T Beutler C Picó AH Shibolet O Pratt DS Pascher A. Enhanced innate immune responsiveness and intolerance to intestinal endotoxins in human biliary epithelial cells contributes to chronic cholangitis. Liver Int. 2011;31:1574–1588. 22093333 10.1111/j.1478-3231.2011.02635.x

[119] Kummen M Holm K Anmarkrud JA Nygård S Vesterhus M Høivik ML. The gut microbial profile in patients with primary sclerosing cholangitis is distinct from patients with ulcerative colitis without biliary disease and healthy controls. Gut. 2017;66:611–619. 26887816 10.1136/gutjnl-2015-310500

[120] Sabino J Vieira-Silva S Machiels K Joossens M Falony G Ballet V. Primary sclerosing cholangitis is characterised by intestinal dysbiosis independent from IBD. Gut. 2016;65:1681–1689. 27207975 10.1136/gutjnl-2015-311004 PMC5036217

[121] Bajer L Kverka M Kostovcik M Macinga P Dvorak J Stehlikova Z. Distinct gut microbiota profiles in patients with primary sclerosing cholangitis and ulcerative colitis. World J Gastroenterol. 2017;23:4548–4558. 28740343 10.3748/wjg.v23.i25.4548 PMC5504370

[122] Little R Wine E Kamath BM Griffiths AM Ricciuto A. Gut microbiome in primary sclerosing cholangitis: A review. World J Gastroenterol. 2020;26:2768–2780. 32550753 10.3748/wjg.v26.i21.2768 PMC7284173

[123] Kummen M Thingholm LB Rühlemann MC Holm K Hansen SH Moitinho-Silva L. Altered gut microbial metabolism of essential nutrients in primary sclerosing cholangitis. Gastroenterology. 2021;160:1784–1798. 33387530 10.1053/j.gastro.2020.12.058 PMC7611822

[124] Schrumpf E Kummen M Valestrand L Greiner TU Holm K Arulampalam V. The gut microbiota contributes to a mouse model of spontaneous bile duct inflammation. J Hepatol. 2017;66:382–389. 27720803 10.1016/j.jhep.2016.09.020 PMC5250551

[125] Tabibian JH O'hara SP Trussoni CE Tietz PS Splinter PL Mounajjed T. Absence of the intestinal microbiota exacerbates hepatobiliary disease in a murine model of primary sclerosing cholangitis. Hepatology. 2016;63:185–196. 26044703 10.1002/hep.27927 PMC4670294

[126] Nakamoto N Sasaki N Aoki R Miyamoto K Suda W Teratani T. Gut pathobionts underlie intestinal barrier dysfunction and liver T helper 17 cell immune response in primary sclerosing cholangitis. Nat Microbiol. 2019;4:492–503. 30643240 10.1038/s41564-018-0333-1

[127] Kellermayer R Carbone M Horvath TD Szigeti RG Buness C Hirschfield GM. Identifying a therapeutic window of opportunity for people living with primary sclerosing cholangitis: Embryology and the overlap of inflammatory bowel disease with immune-mediated liver injury. Hepatology. 2024. Epub ahead of print. doi: 10.1097/HEP.0000000000000926 38743006

[128] Ponsioen CY Chapman RW Chazouillères O Hirschfield GM Karlsen TH Lohse AW. Surrogate endpoints for clinical trials in primary sclerosing cholangitis: Review and results from an International PSC Study Group consensus process. Hepatology. 2016;63:1357–1367. 26418478 10.1002/hep.28256

[129] Boonstra K Weersma RK van Erpecum KJ Rauws EA Spanier BW Poen AC. Population-based epidemiology, malignancy risk, and outcome of primary sclerosing cholangitis. Hepatology. 2013;58:2045–2055. 23775876 10.1002/hep.26565

[130] Villard C Friis-Liby I Rorsman F Said K Warnqvist A Cornillet M. Prospective surveillance for cholangiocarcinoma in unselected individuals with primary sclerosing cholangitis. J Hepatol. 2023;78:604–613. 36410555 10.1016/j.jhep.2022.11.011

[131] LaRusso NF Wiesner RH Ludwig J MacCarty RL Beaver SJ Zinsmeister AR. Prospective trial of penicillamine in primary sclerosing cholangitis. Gastroenterology. 1988;95:1036–1042. 3410217 10.1016/0016-5085(88)90180-1

[132] Angulo P Batts KP Jorgensen RA LaRusso NA Lindor KD. Oral budesonide in the treatment of primary sclerosing cholangitis. Am J Gastroenterol. 2000;95:2333–2337. 11007238 10.1111/j.1572-0241.2000.02323.x

[133] Allison MC Burroughs AK Noone P Summerfield JA. Biliary lavage with corticosteroids in primary sclerosing cholangitis. A clinical, cholangiographic and bacteriological study. J Hepatol. 1986;3:118–122. 3528278 10.1016/s0168-8278(86)80155-6

[134] van Hoogstraten HJ Vleggaar FP Boland GJ van Steenbergen W Griffioen P Hop WC. Budesonide or prednisone in combination with ursodeoxycholic acid in primary sclerosing cholangitis: A randomized double-blind pilot study. Belgian-Dutch PSC Study Group. Am J Gastroenterol. 2000;95:2015–2022. 10950051 10.1111/j.1572-0241.2000.02267.x

[135] Giljaca V Poropat G Stimac D Gluud C. Glucocorticosteroids for primary sclerosing cholangitis. Cochrane Database Syst Rev. 2010;2010:004036. 10.1002/14651858.CD004036.pub3 PMC7163281 20091555

[136] Schramm C Schirmacher P Helmreich-Becker I Gerken G zum Büschenfelde KH Lohse AW. Combined therapy with azathioprine, prednisolone, and ursodiol in patients with primary sclerosing cholangitis. A case series. Ann Intern Med. 1999;131:943–946. 10610645 10.7326/0003-4819-131-12-199912210-00006

[137] Boberg KM Egeland T Schrumpf E. Long-term effect of corticosteroid treatment in primary sclerosing cholangitis patients. Scand J Gastroenterol. 2003;38:991–995. 14531538 10.1080/00365520310005172

[138] Bowlus CL Arrivé L Bergquist A Deneau M Forman L Ilyas SI. AASLD practice guidance on primary sclerosing cholangitis and cholangiocarcinoma. Hepatology. 2023;77:659–702. 36083140 10.1002/hep.32771

[139] European Association for the Study of the Liver. EASL Clinical Practice Guidelines on sclerosing cholangitis. J Hepatol. 2022;77:761–806. 35738507 10.1016/j.jhep.2022.05.011

[140] Knox TA Kaplan MM. A double-blind controlled trial of oral-pulse methotrexate therapy in the treatment of primary sclerosing cholangitis. Gastroenterology. 1994;106:494–499. 8299916 10.1016/0016-5085(94)90610-6

[141] Åberg F Sallinen V Tuominen S Adam R Karam V Mirza D. Cyclosporine vs. tacrolimus after liver transplantation for primary sclerosing cholangitis—A propensity score-matched intention-to-treat analysis. J Hepatol. 2024;80:99–108. 37722533 10.1016/j.jhep.2023.08.031

[142] Van Thiel DH Carroll P Abu-Elmagd K Rodriguez-Rilo H Irish W McMichael J. Tacrolimus (FK 506), a treatment for primary sclerosing cholangitis: Results of an open-label preliminary trial. Am J Gastroenterol. 1995;90:455–459. 7532912 PMC2982698

[143] Talwalkar JA Angulo P Keach JC Petz JL Jorgensen RA Lindor KD. Mycophenolate mofetil for the treatment of primary sclerosing cholangitis. Am J Gastroenterol. 2005;100:308–312. 15667487 10.1111/j.1572-0241.2005.40484.x

[144] Epstein MP Kaplan MM. A pilot study of etanercept in the treatment of primary sclerosing cholangitis. Dig Dis Sci. 2004;49:1–4. 14992426 10.1023/b:ddas.0000011827.87103.2e

[145] Walmsley M Tornai D Cazzagon N Leburgue A Mrzljak A Lenzen H. Patient-reported quality of care in primary sclerosing cholangitis. Liver Int. 2023;43:1654–1662. 37312635 10.1111/liv.15650

[146] Shi J Li Z Zeng X Lin Y Xie WF. Ursodeoxycholic acid in primary sclerosing cholangitis: Meta-analysis of randomized controlled trials. Hepatol Res. 2009;39:865–873. 19467021 10.1111/j.1872-034X.2009.00527.x

[147] Beuers U. Drug insight: Mechanisms and sites of action of ursodeoxycholic acid in cholestasis. Nat Clin Pract Gastroenterol Hepatol. 2006;3:318–328. 16741551 10.1038/ncpgasthep0521

[148] Tse CS Loftus EV Jr. Raffals LE Gossard AA Lightner AL. Effects of vedolizumab, adalimumab and infliximab on biliary inflammation in individuals with primary sclerosing cholangitis and inflammatory bowel disease. Aliment Pharmacol Ther. 2018;48:190–195. 29808485 10.1111/apt.14829

[149] Hedin CRH Sado G Ndegwa N Lytvyak E Mason A Montano-Loza A. Effects of tumor necrosis factor antagonists in patients with primary sclerosing cholangitis. Clin Gastroenterol Hepatol. 2020;18:2295–2304. 32068151 10.1016/j.cgh.2020.02.014

[150] Hommes DW Erkelens W Ponsioen C Stokkers P Rauws E van der Spek M. A double-blind, placebo-controlled, randomized study of infliximab in primary sclerosing cholangitis. J Clin Gastroenterol. 2008;42:522–526. 18344886 10.1097/MCG.0b013e3181662426

[151] Caron B Peyrin-Biroulet L Pariente B Bouhnik Y Seksik P Bouguen G. Vedolizumab therapy is ineffective for primary sclerosing cholangitis in patients with inflammatory bowel disease: A GETAID multicentre cohort study. J Crohns Colitis. 2019;13:1239–1247. 31056693 10.1093/ecco-jcc/jjz088

[152] Lynch KD Chapman RW Keshav S Montano-Loza AJ Mason AL Kremer AE. Effects of vedolizumab in patients with primary sclerosing cholangitis and inflammatory bowel diseases. Clin Gastroenterol Hepatol. 2020;18:179–187. 31100458 10.1016/j.cgh.2019.05.013 PMC6941216

[153] Christensen B Micic D Gibson PR Yarur A Bellaguarda E Corsello P. Vedolizumab in patients with concurrent primary sclerosing cholangitis and inflammatory bowel disease does not improve liver biochemistry but is safe and effective for the bowel disease. Aliment Pharmacol Ther. 2018;47:753–762. 29377235 10.1111/apt.14525 PMC5821055

[154] Shah A Jones MP Callaghan G Fairlie T Ma X Culver EL. Efficacy and safety of biologics in primary sclerosing cholangitis with inflammatory bowel disease: A systematic review and meta-analysis. Hepatol Commun. 2024;8:e0347. 38206197 10.1097/HC9.0000000000000347 PMC10786591

[155] Schregel I Ramos GP Ioannou S Culver E Färkkilä M Schramm C. Evaluation of tofacitinib in primary sclerosing cholangitis and associated colitis: A multicenter, retrospective study. Clin Gastroenterol Hepatol. 2023;21:3448–3450.e3443. 36731589 10.1016/j.cgh.2023.01.014

[156] Chruscinski A Juvet S Moshkelgosha S Renner E Lilly L Selzner N. Autologous hematopoietic stem cell transplantation for liver transplant recipients with recurrent primary sclerosing cholangitis: A pilot study. Transplantation. 2022;106:562–574. 34049362 10.1097/TP.0000000000003829

[157] Voskens C Stoica D Rosenberg M Vitali F Zundler S Ganslmayer M. Autologous regulatory T-cell transfer in refractory ulcerative colitis with concomitant primary sclerosing cholangitis. Gut. 2023;72:49–53. 35428657 10.1136/gutjnl-2022-327075 PMC9763232

[158] Forsyth KS Jiwrajka N Lovell CD Toothacre NE Anguera MC. The conneXion between sex and immune responses. Nat Rev Immunol. 2024;24:487–502. 38383754 10.1038/s41577-024-00996-9 PMC11216897

[159] Mogilenko DA Shchukina I Artyomov MN. Immune ageing at single-cell resolution. Nat Rev Immunol. 2022;22:484–498. 34815556 10.1038/s41577-021-00646-4 PMC8609266

[160] Thome JJ Yudanin N Ohmura Y Kubota M Grinshpun B Sathaliyawala T. Spatial map of human T cell compartmentalization and maintenance over decades of life. Cell. 2014;159:814–828. 25417158 10.1016/j.cell.2014.10.026 PMC4243051

